# The relationship between snoring and left ventricular hypertrophy of China: a cross-sectional study

**DOI:** 10.1186/s12872-016-0185-7

**Published:** 2016-01-15

**Authors:** Naijin Zhang, Ning Ye, Yintao Chen, Xiaofan Guo, Guozhe Sun, Yingxian Sun

**Affiliations:** Department of Cardiology, The First Hospital of China Medical University, 155 Nanjing North Street, Heping District, 110001 Shenyang, Liaoning China

**Keywords:** Snoring, Snoring intensity, Left ventricular hypertrophy, Cardiovascular risk

## Abstract

**Background:**

Population-based investigations studying the association between snoring and left ventricular hypertrophy (LVH) are lacking. Therefore, our study aims to investigate whether snoring is significantly associated with LVH, and to make clear the effect of varying degrees of snoring intensity on LVH.

**Methods:**

A total of 10,139 participants were involved in this cross-sectional study. Snoring status and snoring intensity were evaluated by a structured questionnaire. LVH was defined as left ventricular mass index ≥51 g/m^2.7^ for both men and women.

**Results:**

The total prevalence of LVH was 10.0 %. the prevalence increased significantly in snorers according to snoring intensity, including low (10.3 %), normal (13.1 %), strong (14.7 %) and very strong (16.7 %). After adjustment for age, race, gender, educational status, physical activity, annual income, current smoking status, current drinking status, sleep duration, hypertension, body mass index, waist circumference, low-density lipoprotein cholesterol, high-density lipoprotein cholesterol, triglyceride, total cholesterol and fasting plasma glucose, snoring (OR, 1.371; 95 % CI, 1.147–1.637, *P* < 0.001) was significantly associated with LVH. In addition, among the four kinds of intensity of snoring, normal (OR, 1.436; 95 % CI, 1.126–1.832, *P* = 0.004), strong (OR, 1.462; 95 % CI, 1.124–1.902, *P* < 0.001) and very strong (OR, 1.813; 95 % CI, 1.273–2.684, *P* < 0.001), rather than low (OR, 1.094; 95 % CI, 0.834–1.434, *P* = 0.518) were significantly associated with LVH.

**Conclusions:**

Snoring is independently associated with LVH. What’s more, with the rise in snoring intensity, snoring will exert an increasing effect on LVH.

## Background

Snoring is a loud and jarring noise produced by the joint vibration of tongue and soft palate as a result of the obstructed breathing while sleeping. It is believed to be an important manifestation of obstructive sleep apnea (OSA). Exceptionally, however, still 40 % male snorers and 20 % female snorers do not experience OSA [1, 2]. In addition, lots of studies in recent years showed that snoring was significantly associated with cardiovascular disease (CVD) [3–6].

There are many possible causes to CVD. Studies have verified that snoring is closely linked to metabolic syndrome [7, 8] which has always been considered as an important independent risk factor of CVD [9, 10]. Therefore, it is probable that metabolic syndrome mediates the connection between snoring and CVD. Atherosclerotic plaque is another probable way of mediating the connection between snoring and CVD, for recent studies have verified that snoring is closely associated with atherosclerosis [11, 12].

Having been long considered as an important risk factor of CVD [13, 14], left ventricular hypertrophy (LVH) is closely linked to myocardial infarction, stroke and arrhythmia [15]. Is there possibility that it is by mediating LVH that snoring results in CVD? As far as we know, there hasn’t been any relevant studies to explore whether or not snoring is significantly associated with LVH in the general population. Besides, most previous studies, which estimated the effect of snoring on CVD, always focused on snoring frequency, but few studied the relationship between snoring and CVD by grading the snoring intensity. Hence, our study intend to ascertain whether snoring is significantly associated with LVH, and to make clear the the effect of varying degrees of snoring intensity on LVH.

## Methods

### Study population

The study was conducted from January 2012 to August 2013. A representative sample from the participants aged over 35 years old was selected to present the prevalence, incidence and natural history of cardiovascular risk factors in rural areas of Liaoning Province. The study adopted a multi-stage, stratified, random-cluster sampling scheme. At the first stage, three areas of Dawa, Zhangwu and Liaoyang County were randomly selected from Liaoning province. At the second stage, one town was randomly selected from each county (for a total of three towns). At the third stage, 8 to 10 rural villages from each town were randomly selected (for a total of 26 rural villages). A total of 14,016 satisfactory participants were enrolled in this survey. Among those, 11,956 participants completed this study with a response rate of 85.3 %. And the participants who were pregnant or had malignant tumors or mental disorders or didn’t know whether she/he snoring or missing variables for our multivariable logistic regression models were excluded. Finally, a sample size of 10,139 is accepted. The study was approved by the Ethics Committee of China Medical University, Shenyang, China and all procedures were conducted under ethical standards. Written consent was obtained from all participants after they had been informed of the objectives, benefits, medical items and confidentiality agreement regarding their personal information. For participants who were illiterate, written informed consent were obtained from their proxies.

### Echocardiography measurements

Echocardiograms were performed on each participants by professional sonographers with a commercially available Doppler echocardiograph (Vivid, GE Healthcare, United States), using a 3.0-MHz transducer. Subjects should maintain in the supine position and transthoracic echocardiogram included M-mode, 2-dimensional, spectral and color Doppler. Echocardiogram analyses were performed by three doctors specialized in echocardiography and consultations were made with two other doctors if questions or uncertainty arose. M-mode and two-dimensional images of the left ventricular (LV) wall thickness, internal diameter, aortic root and left atrium was being recorded by eath participants’ parasternal acoustic window. Correct orientation of planes for Doppler recordings and imagings were verified according to previously described procedures [16, 17]. LV internal dimensions (LVID), posterior wall thickness (PWT) and interventricular septal thickness (IVST) were obtained according to American Society of Echocardiography recommendations [17, 18]. Left ventricular mass was calculated according to the formula LVM [19] =0.81 (1.04 [LVID + IVST + PWT]) 3 - (LVID) 3 + 0.06. According to de Simone et al, Left ventricular mass index (LVMI) was normalized for body height in m^2.7^ [20]. LVH was defined as the LVMI ≥51 g/m^2.7^ for both men and women [20].

### Covariate measurements and definitions

Information on covariates, such as demographic characteristics, lifestyle risk factors, family income and family history of chronic diseases, was collected during a single clinic visit by cardiologists and trained nurses using a standard questionnaire by face-to-face interview. Before the survey was performed, we invited all eligible investigators to attend the organized training. The training contents included the purpose of this study, how to administer the questionnaire, the standard method of measurement, the importance of standardization, and the study procedures. A strict test was evaluated after this training, only those who scored perfectly on the test could become investigators. During data collection, our inspectors had further instructions and support.

Race was categorized as Han or others which included some ethnic minorities in China, such as Mongol and Manchu. The questions posed for the educational level were categorized as primary school or below, middle school and high school or above. Sleep duration were self-reported from the participants by asking the following question, ‘How many hours of sleep do you usually have everyday on average (including nocturnal sleep duration and nap duration)? Family income was categorized into three groups ≤5000, 5000–20,000 and >20,000 CNY/year. Current drinking status was defined as one or more alcoholic drinks in the previous year. Current smoking status was defined as a history of 100 or more cigarettes and continued use.

Physical activity which included occupational and leisure-time physical activity, was evaluated by a detailed description [21]. Occupational and leisure-time physical activity were merged and regrouped into 3 categories: 1) low was defined as participants who reported light levels of both occupational and leisure-time physical activity, 2) moderate was defined as participants who reported moderate or high levels of either occupational or leisure-time physical activity, 3) high was defined as participants who reported a moderate or high level of both occupational and leisure-time physical activity.

According to American Heart Association protocol, blood pressure (BP) which was measured three times at two-min intervals after more than 10 min of rest, was measured by a standardized automatic electronic sphygmomanometer (HEM-907; Omron, Japan). Caffeinated beverages and exercise should be avoided for at least 30 min before the measurement. With the arm supported at the level of the heart, the participants were seated appropriately during the measurement. The mean of three blood pressure (BP) measures which was calculated accurately, was used in all analyses. And hypertension was defined as BP ≥140/90 mmHg or currently taking hypertension medication, according to JNC-7 report guidelines [22].

Fasting blood samples were collected after 12 h of fasting in the morning. Blood samples were obtained from an antecubital vein into vacutainer tubes containing ethylenediaminetetraacetic acid (EDTA). Enzymatic reaction was used to analyze blood samples inculding fasting plasma glucose (FPG), high-density lipoprotein cholesterol (HDL-C), low-density lipoprotein cholesterol (LDL-C), triglyceride (TG), total cholesterol (TC) and other routine blood biochemical indexes on an Olympus AU640 autoanalyzer (Olympus, Kobe, Japan). All laboratory equipments were calibrated, and all blinded duplicate samples were used in our study.

### Information on snoring

Snoring status was evaluated by a structured questionnaire, including two questions: 1, Do you know, or someone tell you that you snore? (yes or no or don’t know); and 2, How much is the loudness of sound when you are snoring? (Slightly louder than breathing sound = 1; as loud as speaking = 2; louder than normal speaking = 3; so loud that it can be heard in the next room = 4). According to the question 2, snoring intensity were categorized into low, normal, strong and very strong respectively. These responses were either self-reported or the response of a close relative and this method had been widely used to obtain the information of snoring, especially in epidemiologic studies [3–6, 11, 12].

### Statistical Analysis

Data were expressed as mean ± standard deviation (SD) for continuous variables and numbers (percentages) for categorical variables. Differences in the characteristics of study participants between snorers and non-snorers were determined utilizing the t-test, ANOVA, non-parametric test or the χ2-test, as appropriate. LVH was evaluated with multivariate logistic regression analyses according to snoring and its intensity. Adjustments for potential confounders were as follows: Model 1 = unadjusted; Model 2 = adjusted for age, race, gender, educational status, physical activity, annual income, current smoking status, current drinking status and sleep duration; Model 3 = model 2 plus hypertension, waist circumference, low-density lipoprotein cholesterol, high-density lipoprotein cholesterol, triglyceride, total cholesterol and fasting plasma glucose. Results of logistic regression analyses are reported as odds ratios (ORs) and corresponding 95 % confidence intervals (CIs). All the statistical analyses were calculated using programs available in the SPSS version 22.0 software (IBM Corp., Armonk, NY, USA), and P values were considered to be statistically significant if less than 0.05.

## Results

### Baseline characteristics of participants between snorers and non-snorers

**Fig. 1:**
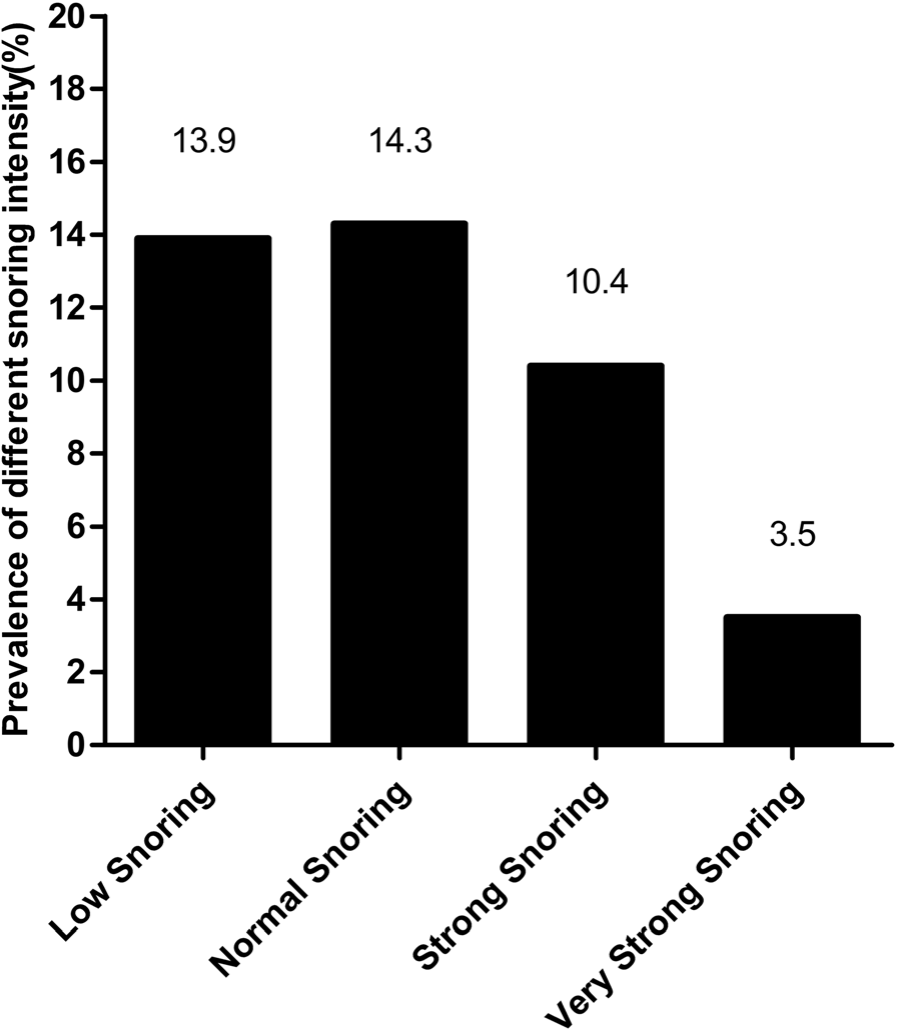
Prevalence of different snoring intensity. The prevalence of each kinds of snoring intensity including low, normal, strong and very strong was 13.9, 14.3, 10.4 and 3.5 % respectively

A total of 10,139 participants were involved in this cross-sectional study. the prevalence of snoring was 42.2 % (*n* = 4275) and the prevalence of four kinds of snoring intensity including low, normal, strong and very strong was 13.9 % (*n* = 1412), 14.3 % (*n* = 1450), 10.4 % (*n* = 1059) and 3.5 % (*n* = 354) respectively (Fig. 1). Compared with non-snorers, snorers were significantly older and had higher levels of systolic blood pressure, diastolic blood pressure, body mass index, height, waist circumference, low-density lipoprotein cholesterol, triglyceride, total cholesterol and the prevalence of current smoking, drinking, diabetes and hypertension (Table 1) (all Ps < 0.05). However, there was no significant difference between the two groups in race, sleep duration and levels of educational status, physical activity and annual income.

**Table 1:** Characteristics of participants according to snoring status

Variables	Non-snorers	Snorers	*P*-value
Mean ± SD			
Age (year)	53.3 ± 10.9	54.5 ± 9.9	<0.001
Male gender (%)	2475 (42.2)	2216 (51.8)	<0.001
Race (Han) (%)	5572 (95.0)	4067 (95.1)	0.793
Educational status (%)			0.065
Primary school or below	2882 (49.1)	2182 (51.0)	
Middle school	2448 (41.7)	1686 (39.4)	
High school or above	534 (9.1)	407 (9.5)	
Physical activity (%)			0.102
Light	1699 (29.0)	1261 (29.5)	
Moderate	3852 (65.7)	2747 (64.3)	
Severe	313 (5.3)	267 (6.2)	
Annual income (CNY/year)			0.662
≤ 5000	732 (12.5)	520 (12.2)	
5000–20000	3161 (53.9)	2343 (54.8)	
> 20000	1971 (33.6)	1412 (33.0)	
Current smoking status (%)	1916 (32.7)	1672 (39.1)	<0.001
Current drinking status (%)	1155 (19.7)	1139 (26.6)	<0.001
Sleep duration (h/d)	7.2 ± 1.7	7.3 ± 1.7	0.069
Systolic blood pressure (mmHg)	138.9 ± 22.8	145.0 ± 23.3	<0.001
Diastolic blood pressure (mmHg)	80.6 ± 11.2	83.8 ± 12.0	<0.001
Height (cm)	160.5 ± 8.0	161.4 ± 8.3	<0.001
Body mass index (kg/m2)	24.1 ± 3.4	25.7 ± 3.8	<0.001
Waist circumference (cm)	80.3 ± 9.3	85.2 ± 9.9	<0.001
Low-density lipoprotein cholesterol (mmol/L)	2.9 ± 0.8	3.0 ± 0.8	<0.001
High-density lipoprotein cholesterol (mmol/L)	1.4 ± 0.4	1.4 ± 0.4	<0.001
Triglyceride (mmol/L)	1.5 ± 1.4	1.8 ± 1.6	<0.001
Total cholesterol (mmol/L)	5.2 ± 1.1	5.3 ± 1.1	<0.001
Fasting plasma glucose (mmol/L)	5.8 ± 1.5	6.0 ± 1.7	<0.001
Diabetes (%)	503 (8.6)	533 (12.5)	<0.001
Hypertension (%)	2648 (45.2)	2482 (58.1)	<0.001

### Echocardiographic characteristics of participants according to snoring status

As the results showed in Table 2, compared with non-snorers, snorers had significantly higher levels of LVID, IVST, PWT, left atrial dimension (LAD), left ventricular mass, left ventricular mass index and prevalence of left ventricular hypertrophy, and lower levels of early/late diastolic peak flow velocity (E/A) (all Ps < 0.05). However, there was no significant difference between the two groups in RWT and ejection fraction (EF).

**Table 2:** Echocardiographic characteristics of participants according to snoring status

Variables	Non-snorers	Snorers	*P*-value
Mean ± SD			
End-diastolic left ventricular internal dimension (LVID), mm	4.7 ± 0.4	4.8 ± 0.4	<0.001
End-diastolic interventricular septum thickness (IVST), mm	0.9 ± 0.3	1.0 ± 0.2	<0.001
End-diastolic posterior wall thickness (PWT), mm	0.9 ± 0.3	1.0 ± 0.2	<0.001
Myocardial relative wall thickness (RWT), mm	0.4 ± 0.1	0.4 ± 0.1	0.074
Left atrial dimension (LAD) (mm)	3.3 ± 0.4	3.4 ± 0.4	<0.001
Ejection fraction (EF), (%)	63.0 ± 3.8	62.9 ± 3.8	0.192
Early/late diastolic peak flow velocity (E/A) ratio	1.1 ± 0.5	1.0 ± 1.5	0.012
Left ventricular mass (g)	137.8 ± 98.3	149.1 ± 80.0	<0.001
Left ventricular mass index (g/m^2.7^)	38.8 ± 27.6	41.0 ± 20.7	<0.001
Prevalence of left ventricular hypertrophy, %	461 (7.9)	551 (12.9)	<0.001

### The prevalence of LVH by different grades of the snoring intensity

**Fig. 2:**
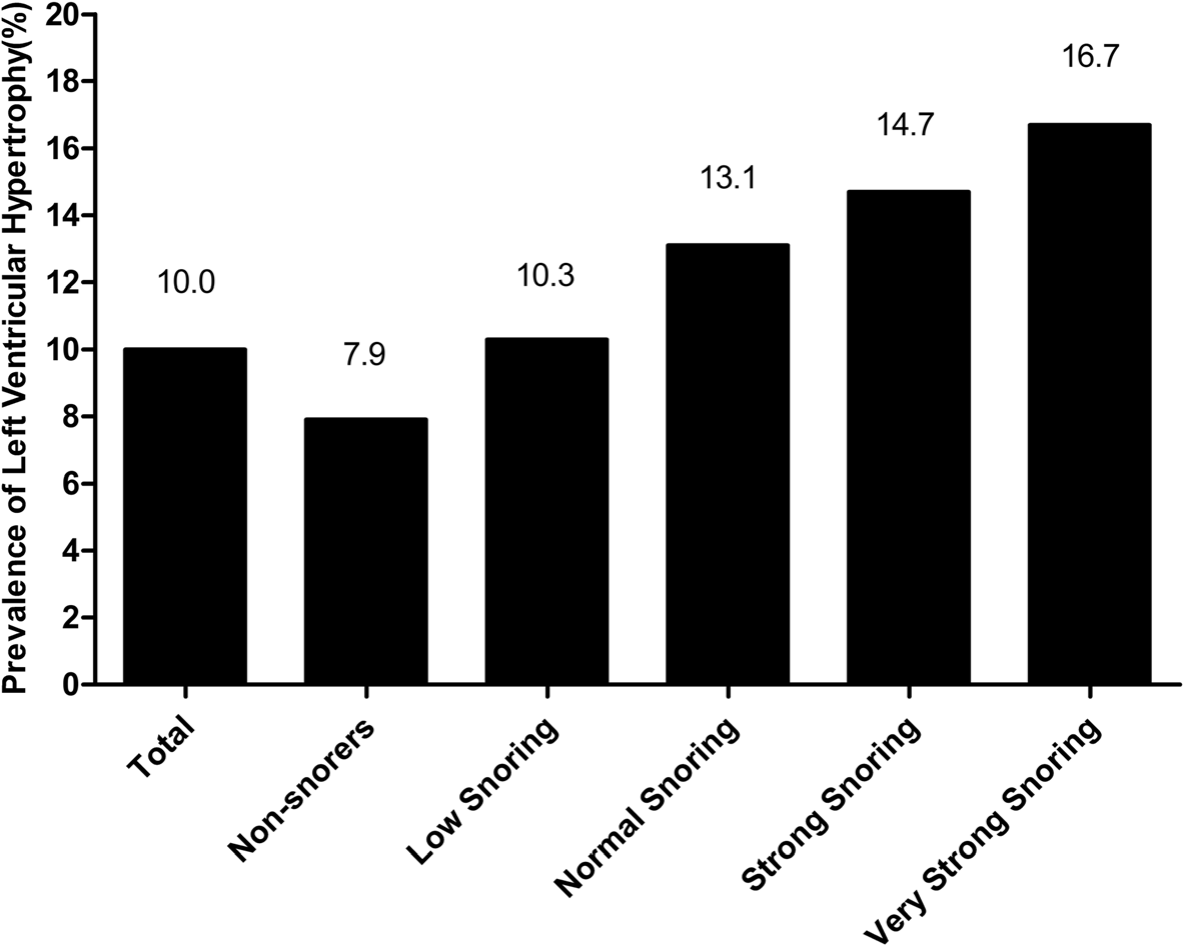
Prevalence of left ventricular hypertrophy by different grades of the snoring intensity. The total prevalence of LVH was 10.0 % and the prevalence of LVH in non-snorers was 7.9 %. The prevalence increased significantly in snorers according to snoring intensity, including low (10.3 %), normal (13.1 %), strong (14.7 %) and very strong (16.7 %). Abbreviations: LVH = left ventricular hypertrophy

The total prevalence of LVH was 10.0 % and the prevalence of LVH in non-snorers was 7.9 %. The prevalence increased significantly in snorers according to snoring intensity, including low (10.3 %), normal (13.1 %), strong (14.7 %) and very strong (16.7 %) (Fig. 2).

In Table 3, we estimated the ORs between snoring and LVH. Unadjusted in model 1, snoring (OR, 1.734; 95 % CI, 1.522–1.976, *P* < 0.001) was significantly associated with LVH. Among the four kinds of intensity of snoring, low (OR, 1.352; 95 % CI, 1.111–1.644, *P* = 0.003), normal (OR, 1.767; 95 % CI, 1.477–2.115, *P* < 0.001), strong (OR, 2.025; 95 % CI, 1.666–2.460, *P* < 0.001) and very strong (OR, 2.344; 95 % CI, 1.745–3.149, *P* < 0.001) were all significantly associated with LVH. After adjustment for age, race, gender, educational status, physical activity, annual income, current smoking status, current drinking status and sleep duration in model 2, snoring (OR, 1.709; 95 % CI, 1.495–1.953, *P* < 0.001) was significantly associated with LVH. In addition, among the four kinds of intensity of snoring, low (OR, 1.381; 95 % CI, 1.131–1.684, *P* = 0.002), normal (OR, 1.774; 95 % CI, 1.476–2.133, *P* < 0.001), strong (OR, 1.886; 95 % CI, 1.545–2.302, *P* < 0.001) and very strong (OR, 2.199; 95 % CI, 1.624–2.977, *P* < 0.001) were all significantly associated with LVH. To farther ascertain the role that metabolic factors play in this relationship, we adjusted model 2 and introduced hypertension, body mass index, waist circumference, low-density lipoprotein cholesterol, high-density lipoprotein cholesterol, triglyceride, total cholesterol and fasting plasma glucose into model 3. Interestingly, still the increased ORs could be observed in group snoring (OR, 1.371; 95 % CI, 1.147–1.637, *P* < 0.001), normal (OR, 1.436; 95 % CI, 1.126–1.832, *P* = 0.004), strong (OR, 1.462; 95 % CI, 1.124–1.902, *P* < 0.001) and very strong (OR, 1.813; 95 % CI, 1.273–2.684, *P* < 0.001) intensity of snoring, rather than low (OR, 1.094; 95 % CI, 0.834–1.434, *P* = 0.518) intensity of snoring. But the ORs were attenuated when we further adjusted for these metabolic factors.

**Table 3:** Multiple regression analyses for the relationship between snoring status, snoring intensity and left ventricular hypertrophy

	Model 1		Model 2		Model 3	
	OR (95 % CI)	*P*	OR (95 % CI)	*P*	OR (95 % CI)	*P*
Snoring status						
Non-snorers	1.000 (reference)		1.000 (reference)		1.000 (reference)	
Snorers	1.734 (1.522–1.976)	<0.001	1.709 (1.495–1.953)	<0.001	1.371 (1.147–1.637)	<0.001
Snoring intensity						
Non-snorers	1.000 (reference)		1.000 (reference)		1.000 (reference)	
Low	1.352 (1.111–1.644)	0.003	1.381 (1.131–1.687)	0.002	1.094 (0.834–1.434)	0.518
Normal	1.767 (1.477–2.115)	<0.001	1.774 (1.476–2.133)	<0.001	1.436 (1.126–1.832)	0.004
Strong	2.025 (1.666–2.460)	<0.001	1.886 (1.545–2.302)	<0.001	1.462 (1.124–1.902)	<0.001
Very strong	2.344 (1.745–3.149)	<0.001	2.199 (1.624–2.977)	<0.001	1.813 (1.273–2.684)	<0.001

Model 1: unadjusted

Model 2: adjusted for age, race, gender, educational status, physical activity, annual income, current smoking status, current drinking status and sleep duration

Model 3: adjusted for model 1 plus hypertension, body mass index, waist circumference, low-density lipoprotein cholesterol, high-density lipoprotein cholesterol, triglyceride, total cholesterol and fasting plasma glucose

*CI* confidence interval, *OR* Odds ratio

## Discussion

The main findings of this large-scale cross-section study were as follows: Firstly, after adjustment for age, race, gender, educational status, physical activity, annual income, current smoking status, current drinking status and sleep duration, snoring and four kinds of snoring intensity were significantly associated with LVH. Secondly, when we further adjusted for the metabolic factors, snoring, normal, strong and very strong snoring intensity, but not low snoring intensity, were significantly associated with LVH. However, the ORs which evaluate the association between snoring, snoring intensity and LVH were attenuated. This suggests that metabolic factors partly mediate the association between snoring, snoring intensity and LVH. Finally, with the increase in snoring intensity, snoring was increasingly risky to LVH. Also we found that the prevalence of LVH increased significantly with the rise in snoring intensity.

Epidemiologic studies have showed that snoring is significantly associated with the development of CVD [3–6]. However, As far as we know, this is the first study to show that snoring is signifacantly associated with LVH in the general population, and also the first to probe into the effect of varying degrees of snoring intensity on CVD. The biological mechanisms that link snoring to LVH remain to be fully elucidated, but some of mechanisms have been proposed. we know that some of the snorers are often accompanied by OSA. It has been verified in relevant studies that OSA constitutes an important risk factor of ventricular remodeling [23, 24]. OSA can reduce the oxygen saturation indices and cause acute sympathetic activation, hemodynamic change and platelet aggregation, which increase cardiac output and induce ventricular remodeling. This is one of the reasons why snoring can lead to LVH. Previous studies showed particular interest in the relationship between OSA and ventricular remodeling; however, the detection of OSA has to use polysomnography. As a result, only a handful of subjects were involved in the studies, far from being persuasive. Compared with OSA, snoring can be used for questionnaire survey to collect data and suitable for large-scale epidemiological investigations [3–6]. In addition, snorers capture snoring symptoms experienced over time, which cannot be acquired by overnight measurement [11].

Metabolic disorder is another probable way of mediating the association between snoring and LVH. In the present study, compared with non-snorers, snorers appear to be more obese, hypertensive, hyperlipemia and diabetic, and the ORs which evaluate the association between snoring, snoring intensity and LVH were attenuated when we further adjusted for these metabolic factors. This suggests that metabolic disorders partly mediate the association between snoring and LVH. As is known to us that hypertension ranks among the most important risk factors of LVH [25]. What’s more, it has been reported in relevant studies that snoring serves as an important independent risk factor of hypertension [26, 27]. Liang Sun [7] showed that snoring, independent of lifestyle factors, adiposity, inflammatory markers and adipokines in apparently healthy Chinese, was significantly associated with metabolic syndrome. And it has been verified in numerous studies that metabolic syndrome is an important risk factor of LVH. Mats Halldin proved in his study [28] that high blood pressure and WC were independent risk factors for LVH and argued that the LVH might result from the changes in the mediated IGF-1 and IGF-Binding Protein-1. Halldin M [29] proved that it was high blood pressure, abdominal obesity and high glucose levels (only limited to in females) that caused LVH. In view of this, we propose those who have experienced chronic snoring should be careful about LVH. Besides, while treating LVH patients with severe snoring, physicians should give appropriate treatment to the snoring of patients.

Nevertheless, there still remains some limitations in our study. Firstly, as our study is based on cross-sectional designs, it is unable to distinguish between cause and effect. Therefore, we propose to verify our conclusions through relevant cohort studies. Secondly, our data on snoring and snoring intensity were obtained from self-report questionnaires, and some subjects living alone were likely to be unaware of their snoring status. Misclassification of snoring status might have attenuated the connection between snoring and LVH, though those who were unclear of their snoring conditions had been excluded from the questionnaires. Finally, our study aims only at Chinese populations. As a result, the present results may not be applicable to populations from other regions and ethnic groups.

## Conclusion

this large-scale cross-sectional study shows that snoring is independently associated with LVH. What’s more, with the rise in snoring intensity, snoring will exert an increasing effect on LVH.

## Abbreviations

LVH: left ventricular hypertrophy
CVD: Cardiovascular disease
BMI: Body mass index
MetS: metabolic syndrome
WC: Waist circumference
BP: Blood pressure
FPG: Fasting plasma glucose
TC: Total cholesterol
LDL-C: Low-density lipoprotein cholesterol
HDL-C: High-density lipoprotein cholesterol
TG: Triglyceride
LVID: end-diastolic left ventricular internal dimension
IVST: end-diastolic interventricular septum thickness
PWT: end-diastolic posterior wall thickness
RWT: myocardial relative wall thickness
LAD: left atrial dimension
EF: ejection fraction
E/A: early/late diastolic peak flow velocity
LVM: left ventricular mass
LVMI: left ventricular mass index
EDTA: ethylenediaminetetraacetic acid
SBP: Systolic BP
DBP: Diastolic BP
WHO: World Health Organization
OR: Odds ratio
CI: Confidence interval
